# Nicolaas (Nico) Westerhof

**DOI:** 10.1007/s12471-022-01706-7

**Published:** 2022-06-22

**Authors:** J. van der Velden

**Affiliations:** 1 Physiology, Amsterdam UMC location Vrije Universiteit Amsterdam, De Boelelaan 1117, Amsterdam, The Netherlands; 2 Amsterdam Cardiovascular Sciences, Heart failure & arrhythmias, Amsterdam UMC, location Vrije Universiteit Amsterdam, Amsterdam, The Netherlands


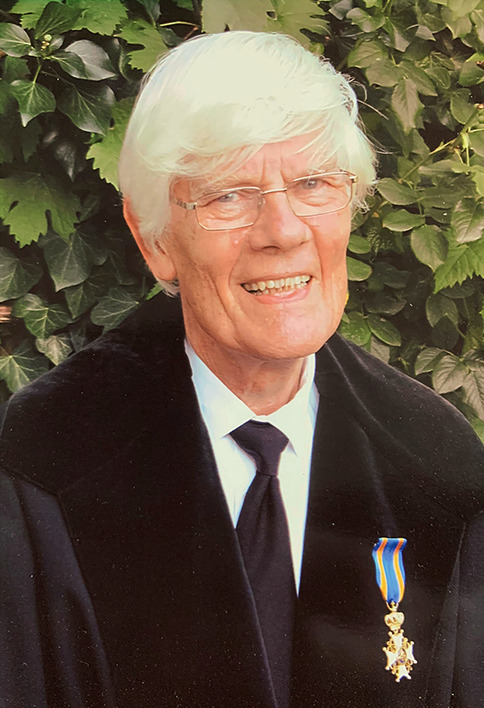


On 25 April 2022, Prof. Nico Westerhof passed away. He studied physics at the University of Utrecht, the Netherlands and received his MSc degree in 1962. From 1964 to 1966, he worked at the Department of Physiology of Georgetown University in Washington, DC, USA, and from 1966 to 1969, he was employed at the Department of Biomedical Engineering, The Moore School of Electrical Engineering at the University of Pennsylvania in Philadelphia, USA. He received his PhD degree from the University of Pennsylvania in 1968.

In 1969, Westerhof was recruited by Prof. Knoop to work at the Department of Physiology of the Medical Faculty of the Free University (*Vrije Universiteit*) Amsterdam, the Netherlands. He worked at the Department of Physiology from 1969 to 2002, where he became a lecturer in 1971 and a full professor in 1980. As emeritus professor, he dedicated his time to mentoring many PhD students at the Department of Pulmonology of the VU University Medical Centre (*VUmc*). His inspiring lectures and enthusiasm for science were highly valued, and despite him being an international leading scientist, it was always easy to approach him, and he was happy to share his thoughts on how to design new experiments and analyse data. Prof. Westerhof’s integrity was undisputed, and he served as a scientific integrity counsellor at the VU University Medical Centre from 2007 to 2014.

Prof. Westerhof’s work on cardiac pump function and the circulation was ground-breaking and at the international forefront. He received international recognition, as illustrated by the many publications in renowned journals, and several awards. In 1996, he received an honorary doctorate from the Ecole polytechnique fédérale de Lausanne, Switzerland. He was also an honorary member of the Italian Society for Experimental Biology and member of the Turin Medical Academy of Sciences. In 2009, he was the recipient of the Dusser de Barenne Coin—a lifetime achievement award—from the Dutch Physiological Society, and in 2011, he received the Lifetime Achievement Award of the ARTERY Society at a meeting in Paris. In 2014, he was appointed Knight in the Order of the Netherlands Lion.

In 1992, he was cofounder of the Cardiovascular Research Institute of the Free University Amsterdam. Under his leadership, the institute became a well-known centre where clinicians and fundamental researchers team up and collaborate to establish bench-to-bedside research. This structure nowadays forms the basis of translational research at the Cardiovascular Research institute at the Amsterdam University Medical Centres. He was president of the Cardiovascular System Dynamics Society from 1996 to 1998 and chairman of the Scientific Advisory Board of the Dutch Heart Foundation from 2003 to 2006.

We will remember Nico as a top scientist, a passionate science-loving person, who was a great lecturer, mentor and coach. I myself was fortunate to have Nico as my doctoral supervisor. He showed me that science has no borders and no hierarchy; his office door was always open. He showed that while being among the brightest persons in the world, one can be modest and kind, and he thereby set the example of what scientific leadership should be.

